# Neonatal polycystic kidney disease, a potential life-threatening condition at this age: A case report

**DOI:** 10.1097/MD.0000000000017707

**Published:** 2019-11-01

**Authors:** Lorena Elena Meliţ, Cristina Oana Mărginean, Cristian Dan Mărginean, Maria Oana Mărginean, Cornel Aldea

**Affiliations:** a Department of Pediatrics I, University of Medicine, Pharmacy, Sciences and Technology Târgu Mureş; b University of Medicine, Pharmacy, Sciences and Technology Târgu Mureş, România; c Department of Pediatric Cardiology, University of Medicine, Pharmacy, Sciences and Technology Târgu Mureş; d University of Medicine and Pharmacy Iuliu Haţeganu Cluj Napoca, Romania.

**Keywords:** newborn, polycystic kidney disease, prognosis

## Abstract

**Rationale:**

Autosomal recessive polycystic kidney disease (ARPKD) is a severe rare genetic condition, with high mortality rates and autosomal recessive pattern of transmission similar to most early onset cystic kidney diseases. The mortality rates can reach up to 30% during the neonatal period.

**Patient concerns:**

We report a case of a 27-day-old male neonate admitted in our clinic for fever, foul-smelling urine, and diarrhea. A previous abdominal ultrasound at the age of 2 weeks revealed enlarged, hyperechoic kidneys, no abnormalities of the urinary exam. Clinical examination revealed poor general status, ill-looking face, diminished cutaneous turgor, distended abdomen, and palpable kidneys. Laboratory tests pointed out leukopenia, anemia, border-line platelet count, elevated inflammatory biomarker level, hyponatremia, hypoalbuminemia, proteinuria, leukocyturia, and hematuria. Both urine and blood cultures were positive for *E. coli*.

**Diagnoses:**

Abdominal ultrasound revealed bilateral nephromegaly, diminished parenchymatous index, with the absence of differentiation between the cortex and medulla. Abdominal MRI described bilateral nephromegaly, the hypertrophy comprising especially the structures of Malpighi pyramids, with multiple cystic lesions disseminated within both kidneys, projected also in Malpighi pyramids, their diameters ranging between 2 and 7 mm. Thus, our final diagnoses were polycystic kidney disease and sepsis due to urinary tract infection with *E. coli*.

**Interventions:**

After treating the infection, the patient was referred to a more experienced center for appropriate management of polycystic kidney disease.

**Outcomes:**

The progress of the patient until the age of 1 year and 2 months has been remarkably favorable, presenting first-degree chronic kidney disease, with normal blood parameters and controlled blood pressure values, no other episodes of urinary infection, and without supplementary pathological changes in ultrasound.

**Lessons:**

Despite the poor prognosis of PKD reported in the literature, our case had an outstandingly favorable evolution during the first 2 years of life most-likely due to the early diagnosis and treatment, but also proper monitoring.

## 1 Introduction

Cystic kidney diseases are defined by the presence of 1 or multiple cysts, which are benign lesions contained in a serous fluid-filled sac.^[1]^ Hereditary and congenital cysts are well-documented to be a result of certain gene mutations that lead to dysfunctions in the primary cilia of the tubular epithelium, whereas the pathogenesis of simple cysts remains unknown.^[2,3]^ The 2 most important and well-known hereditary cystic diseases are autosomal dominant polycystic disease (ADPKD) and autosomal recessive polycystic disease (ARPKD). Similar to other conditions^[4]^, genetic susceptibility is a mandatory condition for both above mentioned disorders, which consist in a diffuse cystic degeneration of the tubular epithelial, resulting in multiple cysts of different diameters.^[1]^ The main differentiation between these 2 disorders is related to the time of diagnosis. Therefore, ADPKD is usually diagnosed during young adulthood, whereas ARPKD is noticed immediately after birth, or even before birth.^[5]^ Moreover, patients with ADPKD have a positive family history and a more benign clinical course compared with patients with ARPKD.^[5]^ Although cases of ARPKD have also been reported in adults, these do not present liver cystic lesions, associating with congenital hepatic fibrosis and/or Caroli disease and only a few macrocysts or not at all.^[6]^

ARPKD is a severe rare genetic condition, with high mortality rates with an autosomal recessive pattern of transmission similar to most early onset cystic kidney diseases.^[7]^ The mortality rates can reach up to 30% during the neonatal period.^[8]^ The incidence of ARPKD is approximately 1:20,000 live births, but it represents the most important cause of end-stage renal disease that requires renal replacement therapy during early childhood.^[9]^ Therefore, up to half of the patients that present this condition seem to develop end-stage kidney disease by the age of 15 years.^[10]^ During the intrauterine life similar to other malformations,^[11,12]^ ARPKD can be detected by ultrasonography with high-resolution probes that will show hyperechoic, increased kidney volume with multiple microcysts within the renal cortex and medulla, with the lack of corticomedullary differentiation.^[13]^ In addition, during the pre- and perinatal periods, the cystic lesions become larger and increase in number.^[1]^ The underlying cause of ARPKD is mutation of the Polycystic Kidney and Hepatic Disease 1 gene (PKHD1), which normally encodes the fibrocystin protein, leading to clinical manifestations since birth, such as enlarged kidneys, progressive renal insufficiency, and arterial hypertension in approximately 80% of the affected infants.^[14]^ In addition, as age increases, liver dysfunction may occur, and the disease can manifest only later in life in certain patients, with a clinical picture dominated by liver complications.^[15]^ If the diagnosis is established during pregnancy, the fetus will present a “Potter” phenotype, consisting of massive kidney enlargement, pulmonary hypoplasia, contracted limbs with club feet, and a characteristic facies. Pulmonary hypoplasia and thoracic compression due to massive enlarged kidneys will result in neonatal death in up to 50% of afflicted newborns.^[5]^ Approximately 80% of the patients diagnosed with ARPKD develop arterial hypertension during the first month of life, which is difficult to control even with multi-drug treatment.^[5]^ Ductal plate malformation due to defective remodeling of the ductal plate, comprising biliary duct ectasia and hepatic fibrosis, will eventually lead to portal hypertension.^[5]^ Moreover, ARPKD represents one of the most important indications for combined liver and kidney transplantation in children.^[5]^ Nevertheless, a small subgroup of patients may display atypical phenotypes consisting of predominant or exclusive impairment of either the liver or kidneys.^[5]^ Although ARPKD is associated with very high mortality rates during the neonatal period, those who survive this period present a 10-year survival rate of approximately 80%.^[10]^

We report a case of a 1-year and 2 month-old male infant who was diagnosed with polycystic kidney disease during the neonatal period to underline the favorable progress despite early onset of this disease and associated complications.

Informed and written consent were obtained from the patient's mother before the publication of this case.

## 2 Case report

### 2.1 Presenting concerns

We report a case of a 27-day-old male neonate who was admitted in our clinic because of fever, foul-smelling urine, and diarrhea with green stools for approximately 1 day. He was previously admitted in our clinic at the age of 2 weeks with the diagnosis of gastro-esophageal reflux. His abdominal ultrasound also revealed enlarged, hyperechoic kidneys, but urine examination showed no abnormalities. Family history did not reveal any pathological elements.

### 2.2 Clinical findings

Clinical examination during admission revealed influenced general status, ill-looking face, diminished cutaneous turgor, genital crisis with edema of the mammary glands, umbilical hernia, distended abdomen, with palpable kidneys. The newborn weighed 4.2 kg.

### 2.3 Diagnostic focus and assessment

**Figure 1:**
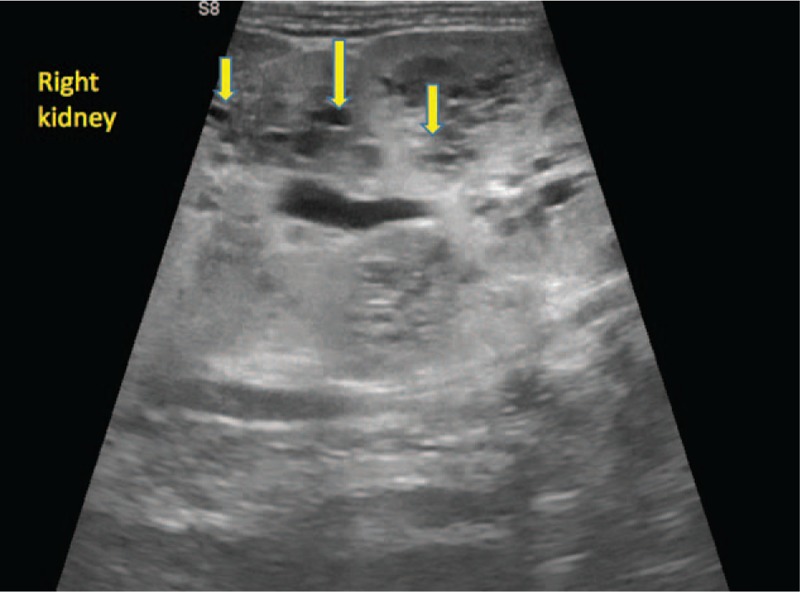
The ultrasound aspect of cystic lesions in the right kidney at the time of diagnosis.

**Figure 2:**
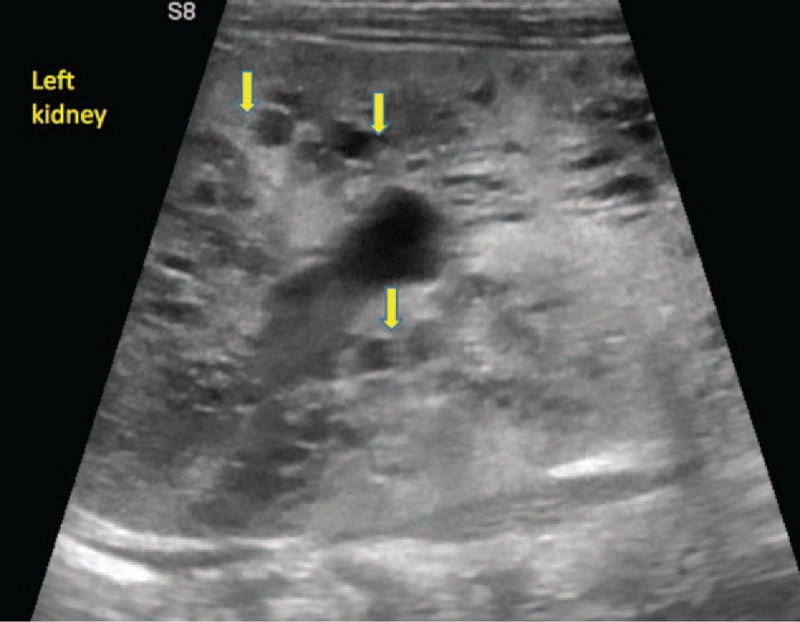
The ultrasound aspect of cystic lesions in the left kidney at the time of diagnosis.

Complete blood count during admission revealed leukopenia (3800/μl), anemia (Hb, 10.7 g/dl; Hct, 30.6%; MCV 84.5 fl; MCH, 29.6 pg), and border-line platelet count (PLT, 160,000/μl). The level of inflammatory biomarkers was severely increased: C-reactive protein (CRP) and erythrocyte sedimentation rate (ESR) were >320 mg/L and 59 mm/h, respectively. Hyponatremia (Na, 117 mmol/L) and hypoalbuminemia (Alb, 2.67 g/dl) were also found. Urinary examination showed 500 proteins/μl, 500 leukocytes/μL, and 300 erythrocytes/μl. Both urine and blood cultures were positive for *Escherichia coli (E. coli),* which proved sensitivity to all of the tested antibiotics (Amikacin, Gentamicin, Amoxicillin/Clavulanic acid, Ampicillin, Cefepime, Cefotaxime, Ceftazidime, Cefuroxime, Ciprofloxacin, Ertapenem, Imipenem, Meropenem, Nitrofurantoin, Norfloxacin, Piperacillin/Tazobactam, Tigecycline, Trimethoprim/Sulfamethoxazole). Abdominal ultrasound revealed bilateral nephromegaly (right kidney, 108.8/54.6 mm; left kidney 100/36 mm), both kidneys showed diminished parenchymatous index, with absence of the differentiation between the cortex and medulla, and multiple transonic oval-shaped masses in the renal parenchyma, with diameter between 2 and 7.5 mm (Figs. 1 and 2). Therefore, we suspected polycystic kidney disease, and abdominal magnetic resonance imaging (MRI) confirmed the diagnosis. Thus, the MRI described bilateral nephromegaly, craniocaudal diameter of the right kidney 110 mm, craniocaudal diameter of the left kidney 111 mm, the hypertrophy comprising especially the structures of Malpighi pyramids, with multiple cystic lesions disseminated within both kidneys, projected also in Malpighi pyramids, their diameters ranging between 2 and 7 mm. The genetic test was postponed due to the parents’ refusal. We established the diagnosis of polycystic kidney disease and sepsis due to urinary tract infection with *E. coli*.

### 2.4 Therapeutic focus and assessment

We initiated empirical treatment with meropenem, and based on the antibiogram result, we decided to continue the therapy with this agent for 10 days until blood and urine parameters were normalized. We also administered human albumin and antipyretics. During the admission, the infant presented elevated blood pressure levels, up to 110/69 mm Hg, and treatment with furosemide, enalapril, and amlodipine was sated based on the recommendations of the cardiologist, but blood pressure levels remained difficult to control. Hyponatremia also persisted. Therefore, we referred the patient to a more experienced center for appropriate treatment.

### 2.5 Follow-up and outcome

**Figure 3:**
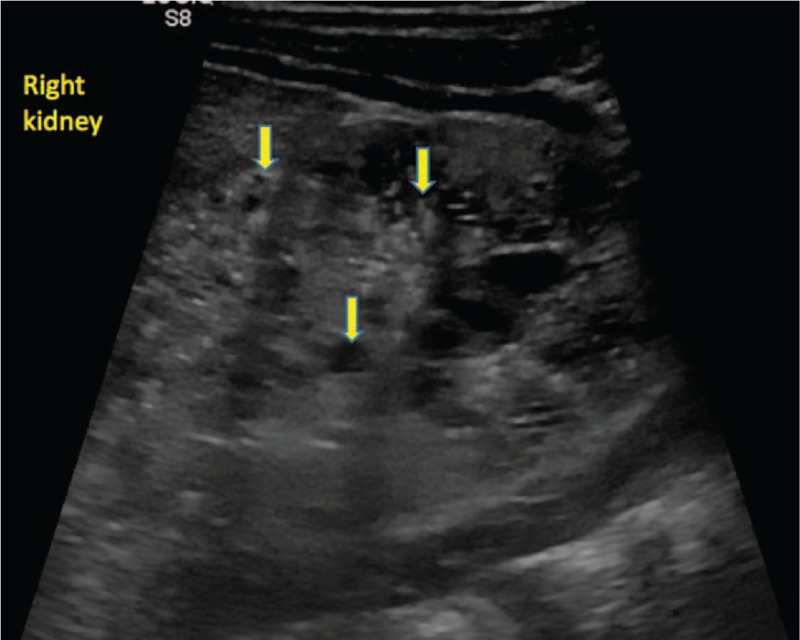
The evolution of cystic lesions in the right kidney (Ultrasound aspect - 1 year and 2 months of age).

**Figure 4:**
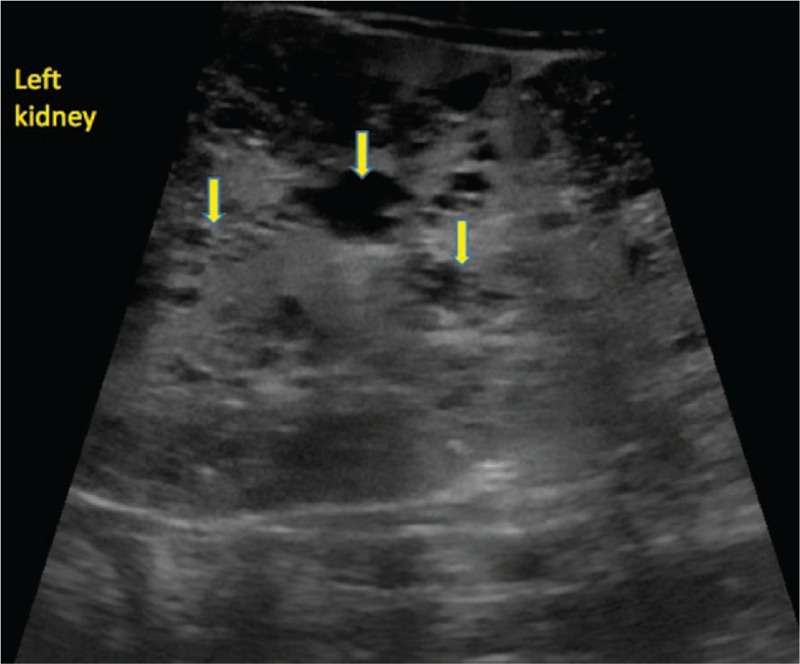
The evolution of the cystic lesions in the left kidney (Ultrasound aspect - 1 year and 2 months of age).

The blood pressure levels were eventually managed by adjusting the doses of amlodipine and enalapril, and by adding metoprolol when needed. For hyponatremia, he was initially administered with intravenous sodium at a dose of 6 mEq/kg/day, and orally afterwards at a dose of up to 1.6 mEq/kg/day along with long-term calcium supplements. Blood transfusion was performed, but erythropoietin was also administered due to the decreasing Hb levels. All laboratory parameters normalized, except for leukocytosis, hematuria, and intermittent albuminuria that persisted on urine examination, but urine culture was negative, and these abnormalities were the result of renal injury. At the age of 1 year and 2 months, the patient presented with first-degree chronic kidney disease. His progress was unexpectedly favorable, with normal blood parameters and controlled blood pressure values. He did not present any episodes of urinary infection, and abdominal ultrasound showed no other supplementary pathological changes (Figs. 3 and 4).

## 3 Discussions

Neonatal polycystic kidney disease is one of the major causes of chronic kidney disease (CKD) at this age.^[16]^ The incidence of CKD at this age is very difficult to establish due to the low number of studies available in the literature. Thus, Wedekin et al reported an incidence of 1 in 10,000 live births in the studied geographic area.^[17]^ Most of the studies available in the literature concern the incidence and etiology of end-stage renal disease, and the reported incidence aged between 0 and 2 years was of 7.1 per million healthy infants with the same age.^[18]^ Based on all these facts, an ultrasound screening program for all newborns in the first month of life would be of real help in order to establish the real incidence of this disorder and to prevent its further complications, similar to other severe disorders.^[19]^ A study on 264 patients from 40 different countries showed that 13% of neonatal end-stage renal disease cases were due to cystic kidney disease, along with congenital anomalies of the kidney and urinary tract (55%), cortical necrosis (11%), and congenital nephrotic syndrome (6%).^[18]^ Fortunately, our patient's evolution was outstandingly favorable, he did not present neonatal end-stage renal disease, developing only first-degree CKD at the age of 1 year and 2 months. Prenatal ultrasound has indeed a major role in the early diagnosis of different malformations,^[11,12]^ such as polycystic kidney disease. Nevertheless, the predictive values of fetal ultrasound are limited because many children detected with major prenatal pathological findings, such as nephromegaly or oligohydramnios, present preserved kidney function after birth.^[9]^ Moreover, prenatal ultrasound is hindered also by maternal or even children's obesity which might influence the adequate visualization of potential associated malformations.^[20–22]^ In our case, all the prenatal routine ultrasound findings were normal. Moreover, the ultrasound performed at the age of 2 weeks, showed only hyperechoic parenchyma without any cysts, suggesting that these cysts increased in size and became obvious between 2 and 4 weeks of life.

Of the 2 main cystic kidney disorders, ARPKD is usually diagnosed during perinatal period, whereas ADPKD is known as adult-type polycystic kidney disease.^[5]^ It is also well-known that most newborns and infants diagnosed with ARPKD do not initially present with liver complications, but these develop further in life.^[15,23]^ In addition, Khan et al showed that despite kidney transplantation, 78% of the patients presented with portal hypertension, and 38% were associated gastrointestinal bleeding.^[24]^ In our case, both liver function parameters and liver ultrasound findings were normal during diagnosis and at the age of 1 year and 2 months. Multi-drug resistant hypertension is also found in patients with ARPKD even during diagnosis.^[5]^ Similarly, our patient developed secondary hypertension that was not initially responsive to therapy, but after he recovered from the sepsis, the blood pressure parameters responded to the antihypertensive treatment. Differential diagnosis of secondary arterial hypertension is essential in children due to the fact that it might be the result of other causes, including different life-threatening intoxications.^[25]^ Angiotensin-converting enzyme inhibitors represent the first-line therapy for this type of hypertension despite the scarcity of available studies, along with calcium-channel blockers, diuretics, and beta-blockers.^[26]^ In case of these patients, the risk of urinary tract infections is high, which require adequate monitoring and early antibiotic treatment to prevent further renal injuries. The sequelae of polycystic kidney disease, involving anemia and electrolyte imbalances, as well as metabolic bone disease, must be properly managed by a pediatric nephrologist.^[26]^ Our patient also benefited from blood transfusion, erythropoietin, and sodium supplementation, along with long-term calcium supplements, being referred to a pediatric nephrologist for a proper monitoring in an experienced health care center. Although ultrasonography remains the most appropriate diagnostic tool for polycystic kidney disease during both pre- and postnatal periods, alternative choices might be abdominal radiography, MRI, and computed tomography scans.^[26]^ Our patient also underwent abdominal MRI, which confirmed the diagnosis.

The prognosis of polycystic kidney disease remains unpredictable. Thus, approximately 30% of the newborns with enlarged, hyperechoic kidneys die within the neonatal period due to pulmonary hypoplasia leading to respiratory distress,^[2]^ and most likely, many of these patients have undiagnosed ARPKD.^[26]^ It is also true that a half of those who survive the neonatal period tend to develop end-stage renal disease within the first decade of their lives, but have 80% survival chance at the age of 10 years.^[10,26]^ Moreover the risk of sepsis is considerably higher in patients with both ARPKD and ADPKD.^[27]^ Although no known therapies prevent cyst formation, complications related to this disorder must be properly monitored and treated to delay progression towards end-stage renal disease as much as possible. Genetic and prenatal counseling, involving important communication and interpersonal skills, must be provided in all cases with a history of neonatal polycystic disease.^[28]^ Thus, the particularities of our cases are especially related to his outstandingly favorable short and long-term evolution. Not only he survived during the neonatal period despite the fact he developed urosepsis with *E. coli*, but he did not develop end-stage renal disease up to the age of 1 year, his condition being fully controlled.

## 4 Conclusions

Neonatal polycystic kidney disease remains a severe condition with high morbidity and mortality rates. Despite the increased mortality rates during the neonatal period, those who survive this period present an increased survival chance due to the advances in the management of this condition. Nevertheless, the prognosis of this condition remains unpredictable due to the wide range of related complications.

## Author contributions

**Conceptualization:** Lorena Elena Meliţ, Cristina Oana Marginean.

**Data curation:** Maria Oana Mărginean, Cornel Aldea.

**Formal analysis:** Cristina Oana Marginean, Cristian Dan Mărginean.

**Investigation:** Lorena Elena Meliţ, Cristina Oana Marginean, Cornel Aldea.

**Methodology:** Cristina Oana Marginean.

**Resources:** Cristina Oana Marginean, Cristian Dan Mărginean.

**Supervision:** Lorena Elena Meliţ, Cristina Oana Marginean.

**Validation:** Lorena Elena Meliţ, Cristina Oana Marginean, Cristian Dan Mărginean, Maria Oana Mărginean.

**Writing – original draft:** Lorena Elena Meliţ, Cristina Oana Marginean, Maria Oana Mărginean.

**Writing – review & editing:** Lorena Elena Meliţ, Cristina Oana Marginean, Maria Oana Mărginean.
